# Insights into the changes in the proteome of Alzheimer disease elucidated by a meta-analysis

**DOI:** 10.1038/s41597-021-01090-8

**Published:** 2021-12-03

**Authors:** Hazal Haytural, Rui Benfeitas, Sophia Schedin-Weiss, Erika Bereczki, Melinda Rezeli, Richard D. Unwin, Xusheng Wang, Eric B. Dammer, Erik C. B. Johnson, Nicholas T. Seyfried, Bengt Winblad, Betty M. Tijms, Pieter Jelle Visser, Susanne Frykman, Lars O. Tjernberg

**Affiliations:** 1 Department of Neurobiology, Care Sciences and Society, Division of Neurogeriatrics, Center for Alzheimer Research, Karolinska Institutet, Solna, Sweden; 2 National Bioinformatics Infrastructure Sweden (NBIS), Science for Life Laboratory, Department of Biochemistry and Biophysics, Stockholm University, S-10691 Stockholm, Sweden; 3 Division of Clinical Protein Science & Imaging, Department of Clinical Sciences (Lund) and Department of Biomedical Engineering, Lund University, Lund, Sweden; 4 Stoller Biomarker Discovery Centre, and Division of Cancer Sciences, School of Medical Sciences, Faculty of Biology, Medicine and Health, The University of Manchester, Manchester Academic Health Sciences Centre, CityLabs 1.0, Nelson Street, Manchester, M13 9NQ UK; 5 Department of Biology, University of North Dakota, Grand Forks, ND USA; 6 Goizueta Alzheimer’s Disease Research Center, Emory University School of Medicine, Atlanta, GA USA; 7 Department of Biochemistry, Emory University School of Medicine, Atlanta, GA USA; 8 Department of Neurology, Emory University School of Medicine, Atlanta, GA USA; 9 Karolinska University Hospital, Theme of Inflammation and Aging, Huddinge, Sweden; 10 Alzheimer Center Amsterdam, Department of Neurology, Amsterdam Neuroscience, Vrije Universiteit Amsterdam, Amsterdam UMC, Amsterdam, The Netherlands; 11 Alzheimer Center Limburg, School for Mental Health and Neuroscience, Maastricht University, Maastricht, The Netherlands

**Keywords:** Alzheimer's disease, Proteomics

## Abstract

Mass spectrometry (MS)-based proteomics is a powerful tool to explore pathogenic changes of a disease in an unbiased manner and has been used extensively in Alzheimer disease (AD) research. Here, by performing a meta-analysis of high-quality proteomic studies, we address which pathological changes are observed consistently and therefore most likely are of great importance for AD pathogenesis. We retrieved datasets, comprising a total of 21,588 distinct proteins identified across 857 postmortem human samples, from ten studies using labeled or label-free MS approaches. Our meta-analysis findings showed significant alterations of 757 and 1,195 proteins in AD in the labeled and label-free datasets, respectively. Only 33 proteins, some of which were associated with synaptic signaling, had the same directional change across the individual studies. However, despite alterations in individual proteins being different between the labeled and the label-free datasets, several pathways related to synaptic signaling, oxidative phosphorylation, immune response and extracellular matrix were commonly dysregulated in AD. These pathways represent robust changes in the human AD brain and warrant further investigation.

## Introduction

Mass spectrometry (MS)-based proteomics is a powerful technique, as it allows a simultaneous identification and quantification of proteins in complex biological samples such as brain tissue. These studies typically use a bottom-up approach in which proteins are first digested, the resulting peptides are then analyzed by liquid chromatography coupled to tandem mass spectrometry (LC-MS/MS), and lastly the generated mass spectra of the peptide ions are compared against the theoretical spectra from databases for protein identification. Two main approaches, labeled or label-free, are commonly used for quantification of the relative abundance of the identified proteins. In the labeling strategies, such as tandem mass tags (TMTs) or isobaric tags for relative and absolute quantification (iTRAQ), stable tags are chemically attached to free amine on the N-termini of the peptides and lysine side-chains^1,2^. The principle of isobaric labeling strategies relies on the fact that labeled peptides have the same overall mass due to the isobaric chemical structure of the tags and therefore are chromatographically indistinguishable, but once the peptides are fragmented by collision-induced dissociation via tandem MS (MS2), the reporter ions are released and used for relative protein quantitation^1^. One advantage of this approach is the ability to label different biological samples with different isobaric tags so that they can be analyzed together in the same LC-MS/MS, thus reducing inter-run variability. One drawback with this approach is that the quantification can be affected by interference from isobaric tags derived from coeluting peptides with similar mass, resulting in an underestimation of the fold change – so called ratio compression^3^. In contrast, in the label-free MS approach, each sample is individually analyzed by LC-MS/MS and relative quantitation is often done using the chromatographic precursor ion peak intensity coming from MS1^4^. Thanks to recent advancements allowing more rapid analysis and thus increased proteome coverage, a more reliable protein quantitation can be achieved by LC-MS/MS. This has led to increased application of MS-based proteomics in clinical research to investigate proteins and pathways underlying disease pathophysiology.

One disease that has been extensively studied in the proteomics field is Alzheimer disease (AD), the most common cause of dementia^5^. Many proteomic studies have focused on identifying changes in the proteome of a single brain region in AD cases compared to control subjects^6–10^ while others investigated regional vulnerability by comparing multiple brain regions from the same cohort^11–13^. In a few studies, after employing laser microdissection techniques, region-^14,15^, cell-^16^ or structure-specific (e.g., amyloid plaques)^17,18^ proteomes have also been investigated. Furthermore, the proteomic changes occurring in a specific brain region over the course of Braak stages has been explored^15,19^. Mounting evidence from proteomic studies has shed light onto a number of disrupted cellular mechanisms such as synaptic signaling, mitochondrial bioenergetics, immune response, RNA homeostasis, lipid metabolism and vesicle trafficking – supporting the notion that AD is a multifactorial disease^6,7,14,19^.

Here, we performed a study-level meta-analysis to identify the most robustly affected proteins and pathways in AD brain across cohorts and studies, as well as to investigate potential reasons that could explain the discrepancies observed in the published proteomic studies. Our findings reveal that individual protein alterations observed in AD were clearly dependent on whether a labeled or label-free MS approach was used. However, comprehensive pathway analysis found the involvement of common pathways related to synaptic signaling, oxidative phosphorylation, immune response and extracellular matrix (ECM) in AD brain, irrespective of the MS quantitative method of choice.

## Results

**Fig. 1:**
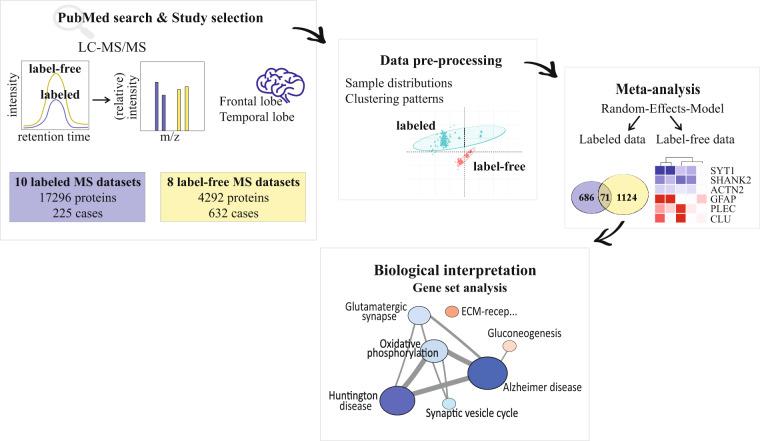
The workflow of the study. After literature search, 18 datasets from ten different MS-based proteomic studies, using either labeled or label-free quantification, were retrieved. These studies were done on postmortem human brain tissue, which were categorized into frontal or temporal lobes, severely affected regions by AD pathology, and consisted of AD (5 ≤ n ≤ 252) and control (5 ≤ n ≤ 94) cases. Subsequently, data pre-processing was done so that datasets would be comparable for further statistical analyses. Sample distributions and the presence of any clustering patterns were assessed using principal component analysis (PCA). The datasets generated by labeled and label-free MS approaches were concatenated separately, and meta-analysis using a random-effects-model was performed. Lastly, for better biological interpretation of our findings, gene set analysis was performed.

Out of 10 studies using labeled (TMT, iTRAQ and ^18^O labeling)^8–12,14^ or label-free^6,7,19^ MS approaches, 18 datasets that analyzed the proteome of frontal and temporal cortices were retrieved. Data containing isoform-specific information was combined by using UniProtKB accession number as a common identifier between studies. This resulted in a dataset with 21,588 distinct protein IDs, 533 of which were identified in all 857 postmortem human samples. Protein intensities from the retrieved datasets were standardized (i.e., allowing 20% missing values per group, log2 conversion, median 0 and standard deviation 1). As source of variance could arise from differences in biological samples and methodology (e.g., instrumentation, database search platforms, application of batch correction or other regression models), a random-effects-model was used to calculate the effect size (Fig. 1).

### Samples of labeled vs label-free datasets appears in distinct clusters

**Fig. 2:**
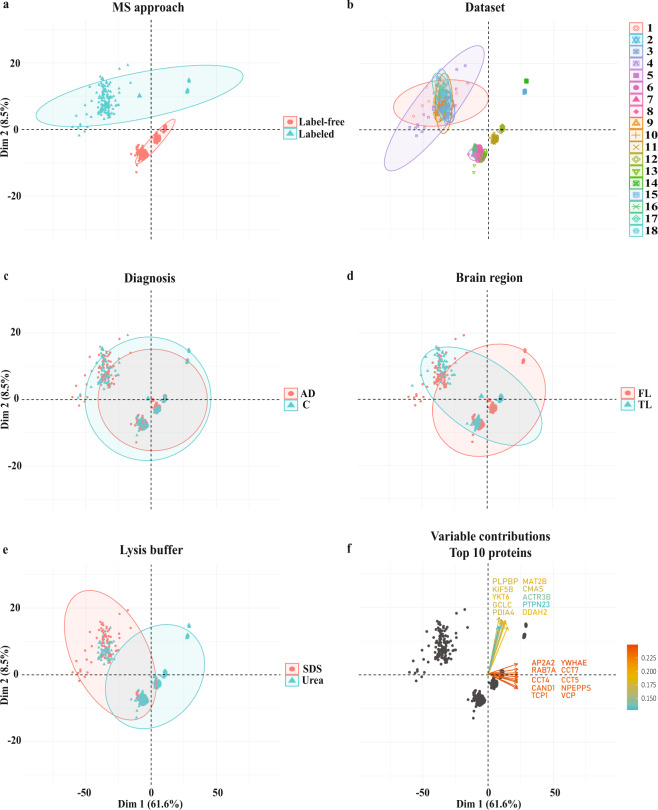
Sample distributions visualized by principal component analysis (PCA). This analysis was performed using log2 intensities of 533 proteins, which were quantified in all 857 (547 AD and 310 control) cases and in all 18 datasets. Sample distributions were grouped by (**a**) MS approach (labeled *vs* label-free quantification), (**b**) datasets, (**c**) diagnosis (AD *vs* control), (**d**) brain region (frontal *vs* temporal lobe), and lastly (**e**) lysis buffer (SDS- *vs* urea-based). PCA showed a clear separation between labeled and label-free MS approaches. (**f**) The top 10 proteins contributing most to component 1 and component 2 were shown respectively. Elipses indicate the 95% confidence interval of samples in each of the groups. Data was standardized prior to PCA.

To investigate the largest variability between samples and to determine whether there were clustering patterns that could be explained by some of the methodological differences among the selected studies, principal component analysis (PCA) was performed using the log2 protein intensities of 533 proteins quantified in all 857 samples (547 AD and 310 control cases). Two main clusters pertaining to labeled or label-free MS approaches (Fig. 2a) suggested that relative protein intensities were specific to the MS method of choice, which may partly be explained by an underestimation of the fold change in the labeled samples due to ratio compression. No clear separation was observed between datasets (Fig. 2b), diagnosis (AD *vs* control, Fig. 2c), brain regions (frontal *vs* temporal lobes, Fig. 2d) or lysis buffer (SDS- *vs* urea-based, Fig. 2e). The top 10 proteins contributing to the first (PC1) and second (PC2) components are also shown in Fig. 2f. Intriguingly, two datasets from Bai *et al*.^9^ were clustered further away from all other labeled datasets. While this cannot be explained by the choice of labeling approach, it is possible that differences in methodology (e.g., cell-type correction) could result in this disparity^9^.

### Differences and similarities between the labeled and the label-free datasets

**Fig. 3:**
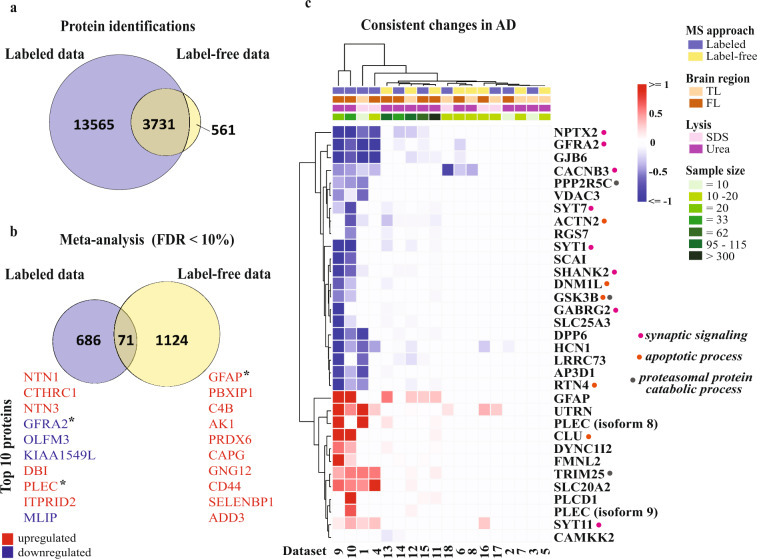
Summary of the meta-analysis findings. Venn diagrams showing (**a**) the total number of identified proteins (with distinct protein IDs) shared between the labeled and the label-free datasets, and (**b**) the statistically significant proteins with FDR < 10% identified by the meta-analysis of the labeled and the label-free datasets. Despite a large overlap found between the two data subsets (3731 proteins), only 71 proteins were significantly altered in AD in both meta-analyses. The top 10 most significantly altered proteins were shown next to the Venn diagram. *These proteins were among the shared proteins. (**c**) Out of 71 significantly altered proteins, 33 were identified as the most robust changes in AD, since the direction of change remained the same across labeled and label-free datasets. Some of these proteins were found to be involved in synaptic signaling, apoptotic and proteasomal protein catabolic processes. Proteins that showed no statistically significant difference between AD and controls as well as the ones that were not identified in the original dataset are indicated by the white boxes.

Based on the distinct sample distributions observed above (Fig. 2a), we performed separate meta-analyses for labeled and label-free datasets. The labeled data consisted of 17,296 distinct proteins quantified across 225 (123 AD and 102 control) cases while the label-free data contained 4,292 distinct proteins quantified across 632 (424 AD and 208 control) cases (Online-only Table 1). When comparing the total protein identifications between the labeled and the label-free data, 3,731 proteins were found to be shared (Fig. 3a). The input data that was used for meta-analyses can be found at the figshare database^20^.

The meta-analysis highlighted that 757 (391 down- and 366 upregulated) proteins were significantly altered in AD in the labeled data (p-value < 0.005 and false discovery rate (FDR) <10%), compared to 1,195 (634 down- and 561 upregulated) proteins in the label-free data (p-value < 0.03 and FDR < 10%) (Fig. 3b). The findings of both meta-analyses can be found at the figshare database^20^. Notably, among significant alterations, 604 proteins (80%) were originally quantified in two or more labeled datasets and 1038 proteins (87%) were quantified in more than one label-free dataset^20^.

Despite the large number of shared proteins between the labeled and the label-free datasets (Fig. 3a), only 71 proteins (1.9%) were found to be significantly altered in AD in both datasets (Fig. 3b). Of these, 33 proteins (46%) showed consistent alterations in AD, in other words the mean difference between AD and control, computed by meta-analyses, was in line with the fold changes found in individual studies (Fig. 3c). Several of these proteins were found to be implicated in synaptic signaling pathways, apoptotic signaling and proteasomal protein catabolic processes. Importantly, our analysis led to the identification of novel proteins involved in AD (Fig. 3c), such as GDNF family receptor alpha-2 (GFRA2), voltage-dependent L-type calcium channel subunit beta-3 (CACNB3), utrophin (UTRN), sodium-dependent phosphate transporter 2 (SLC20A2) and synaptotagmin-11 (SYT11).

### Gene set analysis (GSA) identifies common pathways in the meta-analysis of the labeled and the label-free data

To put single protein alterations into biological context, we performed GSA^21^. All proteins (in gene-centric format) were subjected to GSA (for input data, see^20^), despite that some of them were originally quantified only in one dataset or had opposite directional changes between meta-analyses and individual studies.

**Fig. 4:**
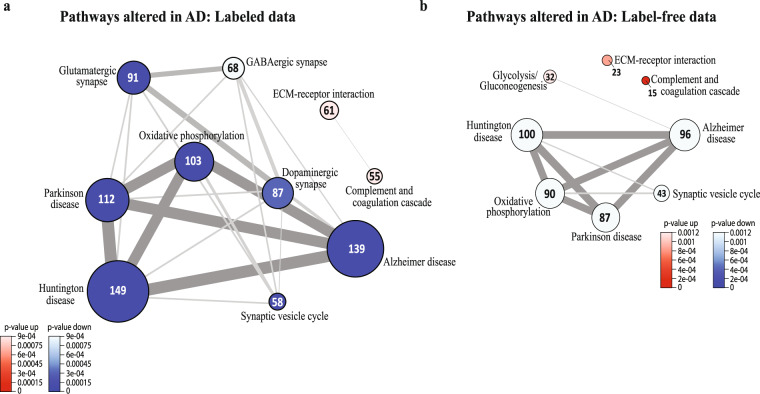
Summary of the gene set analysis. The network plots show examples of the significantly altered pathways from KEGG database (FDR < 5%) in the (**a**) labeled and (**b**) label-free datasets.

By using the KEGG 2019 database, GSA showed that synaptic signaling (e.g., synaptic vesicle cycle), oxidative phosphorylation and pathways related to neurodegenerative disorders, such as AD and Huntington disease (HD), were significantly downregulated in AD in the labeled data (FDR < 5%, Fig. 4a). In addition, complement and coagulation cascade and ECM-receptor interaction pathways were significantly upregulated in AD in the labeled data. Similar changes in biological processes were detected in the label-free data, with the exception that the glycolysis/gluconeogenesis pathway was significantly upregulated in AD (Fig. 4b). Similarly, using the GO biological process database, GSA showed pathways related to synaptic signaling, mitochondrial metabolism, ECM organization and immune response, thereby greatly strengthening the notion that these pathways are involved in disease pathogenesis. All GSA findings are deposited in the figshare database^20^.

## Discussion

We performed a meta-analysis of proteomic studies with the aim of identifying proteins and pathways that are robustly related to AD pathogenesis as well as elucidating discrepancies between the studies. Three different labeling techniques were employed in the original studies including TMT, ^18^O and iTRAQ. Since sample distributions pertaining to these techniques showed a high degree of overlap in the PCA plot, we together call them labeled datasets. Particularly, by comparing the meta-analysis of the labeled and the label-free MS datasets separately, our study provides further knowledge on how the observed proteome might change depending on the MS method of choice. The notion that these two MS approaches rely on different protein quantification methods^22^, it is not surprising that PCA showed a clear separation, however we cannot rule out that other factors might also contribute to this clustering pattern.

Our meta-analysis findings show that the observed protein alterations were clearly different between the two techniques, since only 71 significantly altered proteins (FDR < 10%) were shared. Several reasons may explain why these 71 proteins stood out from the other significant alterations: they are robustly quantified independent of MS approach or they are not sensitive to discrepancies in sample preparation procedures. To get a better understanding of these 71 significantly altered proteins, we further considered whether the mean difference (i.e., the main outcome of meta-analysis) and the fold changes found in each individual dataset followed the same direction, i.e., the protein was either upregulated or downregulated across datasets. We found 22 consistently downregulated and 11 consistently upregulated proteins emerging as the most robust changes occurring in AD brain (Fig. 3b). Importantly, by performing this large dataset analysis, we were able to find novel proteins involved in AD, some of which were involved in synaptic or cell adhesion pathways, which will be discussed below. For instance, we detected upregulation in SLC20A2, which is involved in phosphate transport by absorbing phosphate from interstitial fluid. Interestingly, mutations in this gene are reported to cause primary familial brain calcification, which are often associated with movement disorders^23^. In addition, our findings validate the previously reported data on AD-related proteins. For instance, the astrocytic marker glial fibrillary acidic protein (GFAP) is well-known to be upregulated in AD brain as part of concurrent gliosis. Clusterin (CLU) is identified as a genetic risk factor for sporadic AD^24,25^. Compelling evidence suggests that CLU binds to the amyloid β-peptide (Aβ) and affects its deposition and clearance (see review by Foster *et al*.^26^). Interestingly, Wojtas *et al*. recently demonstrated that CLU not only affects Aβ pathology^27^ but also tau pathology^28^. Another interesting protein from the heatmap is the mitochondrial protein DNM1L (also known as DRP1) which is vital for mitochondrial fission events. Several studies reported a relationship between Aβ-mediated toxicity, DRP1 function and mitochondrial dysfunction^29,30^. In addition, a recent study suggested that DRP1 is required for proper synaptic function in CA1 hippocampal neurons^31^. Several proteins related to synaptic signaling were consistently altered in AD brain across datasets. Among them, synaptotagmin-1 (SYT1) and synaptotagmin-7 (SYT7), acting as calcium sensors triggering neurotransmitter release, are reported to interact with amyloid precursor protein (APP) and presenilin 1 (PS1), a component of γ-secretase that mediates the final cut in APP processing^32–35^. Hyperpolarization-activated cyclic nucleotide-gated channel 1 (HCN1), important for regulating neuronal activity, has been studied extensively in epilepsy research but not much in AD research. However, decreased levels of HCN1 were in line with our finding observed in AD brain, and loss of HCN1 gene function was reported to increase Aβ levels in mice brain^36^. Recent studies identified dipeptidyl aminopeptidase-like protein 6 (DPP6), which modulates the activity of potassium channels, as a novel genetic factor contributing to dementia^37,38^. Functional studies suggested that DPP6 is important for synaptic structure, hippocampus-dependent learning, and memory^39^. Our meta-analysis confirms that DPP6 is of importance for AD pathogenesis and merits further studies.

While the observations on an individual protein level are interesting, we sought to test whether and how the meta-analyses of labeled and label-free datasets would show similar patterns in a wider perspective. Notably, our findings indicate that common biological processes were dysregulated in AD brain, even though the individual protein alterations detectible were clearly different between the two MS approaches. For instance, synaptic processes, such as synaptic vesicle cycle (KEGG hsa04721) and chemical synaptic transmission (GO:0007268), were significantly downregulated in AD. This is not surprising considering that synaptic dysfunction occurs early in AD pathogenesis and strongly correlates with cognitive decline^40^. Examples of synaptic proteins involved in these processes included components of the adaptor protein complex 2 (AP2A2 and AP2M1), disk large-associated protein 1 (DLGAP1), glutamate receptors (GRIA2–4 and GRM3), neuronal pentraxin-2 (NPTX2), RAB3A, SNAP25, SYT1 and V-ATPase subunit F (ATP6V1F). As illustrated by the heatmap, the synaptic proteins NPTX2 and SYT1 were consistently decreased in AD brain across studies, representing one of the most robust changes. Interestingly, AP2A2, involved in clathrin-dependent endocytosis, was among the proteins contributing most to the clustering pattern observed on PCA plot. Many studies have reported reduction in synaptic protein expression (e.g., SNAP25, GRIA2) in AD brain and shown that such decrease was correlated with increased rate of cognitive decline^8,41,42^. Besides the above-mentioned synaptic proteins, we also detected robust downregulation in the levels of GFRA2, which is a receptor for the neurotrophic factor called neurturin. A recent genome-wide association study identified the GFRA2 locus as a potential modifier risk of frontotemporal dementia, proposing that GFRA2-related processes may hold a potential as therapeutic targets^43^. Unlike other synaptotagmins, SYT11 does not bind to calcium while mediating vesicle trafficking^44^, and it is also interesting that SYT11 is the only upregulated synaptic proteins in our meta-analyses.

Another prominently downregulated pathway was related to oxidative phosphorylation (KEGG hsa00190) and mitochondrial energy metabolism. Mainly proteins of the electron transport chain (e.g., NDUFS1, NDUFS7, NDUFA7) were associated with these pathways. Altered mitochondrial dynamics and bioenergetic metabolism are commonly observed in neurodegenerative disorders^45^. This is not surprising since activities such as synaptic transmission and synaptic vesicle cycle demand high metabolic energy that is tightly regulated by mitochondria^46^.

It is noteworthy that HD was the pathway with highest number of protein hits. In this regard, it is interesting to note that our previous immunohistochemical studies have shown increased levels of huntingtin in pyramidal neurons both in AD brain^47^ and in an AD mouse model (APP-NLF)^48^. Many of the altered proteins were mitochondrial, particularly those involved in the respiratory chain. There were also significant effects on proteins involved in clathrin-mediated endocytosis, and for instance postsynaptic receptors and signaling molecules. These findings support that HD and AD partially share disease pathways involved in mitochondrial function, clathrin-mediated endocytosis, postsynaptic function and signaling.

Several pathways were found to be significantly upregulated in AD, such as complement and coagulation cascade (KEGG hsa04610), cytokine-mediated signaling (GO:0019221), which reflects the presence of neuroinflammation, ECM-receptor interaction (KEGG hsa04512) and ECM organization (GO:0030198), indicating changes in the ECM. The ECM provides structural support that is essential for the cells and regulates many cellular processes such as neurogenesis, axonal outgrowth, synaptic plasticity, and immune response^49^. In line with previous findings^15,50^, we mainly detected upregulation of the ECM components, e.g., collagen, CD44, tenascin, integrin alpha-6, versican core protein and fibronectin-1. In addition, we detected robustly increased levels of UTRN (or dystrophin-related protein 1), which mediates the interaction between the plasma membrane, the cytoskeleton and the ECM.

Our study is the largest meta-analysis study analyzing proteomic data from multiple research centers. Thus far, only a few studies compared proteomic datasets in a systematic manner and identified several proteins as promising targets for maintenance of cognitive resilience^42,51^. Similar to the observations from Wingo *et al*.^42^, we found decreased levels of synaptic (e.g., GRIA2, AP2A2, AP2B1, BAIAP2, DMXL2, DLG4, SYNPO) and mitochondrial proteins (e.g., NDUFS1, PDHA1) but increased levels of proteins involved in myelination and apoptosis processes (e.g., GFAP, GSN, NEFL) in AD brain. Altogether these findings corroborate the involvement of these proteins and processes in disease pathogenesis.

Considering that proteomics data extensively relies on experimental setups such as sample preparation^52^, clinical characteristics of subjects, statistical tests of choice or even cut-offs for statistical significance, a meta-analysis could provide valuable and coherent information that a single dataset analysis could not. However, this study is not without challenges. From a statistical perspective, the random-effects-model could compute the mean difference between AD and control groups, even when a given protein was originally quantified in only one dataset or when proteins had originally opposite directional changes. This is possible because the statistical model gives larger weight to the datasets that contain larger effect size, larger sample size, and lower within-study variability. With that said, from a biological perspective, it is important and relevant to pinpoint proteins with consistent alterations across individual datasets, as they could represent the most robust alterations associated with disease. In this study, we address both perspectives by reporting all significantly altered proteins in the labeled (n = 757) and the label-free (n = 1,195) datasets as well as the robustly altered ones (n = 33). It is important to note that our study does not allow any direct comparison between labeled and label-free MS approaches, since the same biological samples were not studied using both methods. However, the observation of two distinct clusters pertaining to labeled and label-free datasets provided good reason for our stratification wherein we performed two different meta-analyses. At the protein level, our findings suggest that different sets of proteins were significantly altered in the labeled dataset compared to the label-free dataset. It is thus important to emphasize that this could be influenced by how each individual study has dealt with protein inference, which could overestimate the number of distinct protein IDs as well as underestimate commonalities between the datasets. We also noted that the total number of proteins (17,296 *vs* 4,294 proteins) and sample size (225 *vs* 632 cases) were rather different between the two methods, which could influence the meta-analysis outcome. Nevertheless, we observed consistent alterations at the pathway level. The analysis of raw MS data in a common pipeline, using the same database search engines for protein identification and quantification, could dissipate some of the methodological differences between the studies, however, this is not without challenges especially when applied to such quantities of data.

Given the complex and multifactorial nature of AD, disentangling pathological mechanisms is of the utmost importance for development of treatment strategies for this debilitating disorder. This study provides a comprehensive analysis of 18 proteomic datasets and offers novel insights into single protein alterations related to AD. Depending on the MS method of choice (labeled *vs* label-free), different proteins appeared to be significantly dysregulated in AD. Nonetheless, pathway analyses of labeled and label-free MS datasets illustrated that processes related to synaptic signaling, oxidative phosphorylation, immune response and ECM were commonly dysregulated in AD. These observations are largely in line with previous reports, indicating that these mechanisms are central to AD pathogenesis.

## Methods

### Study selection and search strategy

Literature search on MS-based proteomic studies in AD was done using the following string “Alzheimer* AND proteome AND (proteomic OR mass spectrometry) AND “human brain”” on April 2, 2020 in PubMed (https://pubmed.ncbi.nlm.nih.gov/). PRISMA statement was followed during study design^53^. Out of 95 articles, 16 datasets were manually retrieved and curated from high-quality studies performed in seven different research groups^6–12,14,19^ (Online-only Table 1), based on the following criteria: (i) sample size of at least five cases per diagnosis group (AD and control), (ii) minimum number of 1000 quantified proteins, (iii) studies in which quantified protein intensities were reported for each individual biological sample, (iv) studies that are publicly available in repositories, and (v) studies in which the proteome of frontal and temporal cortices, severely affected regions by AD-related pathology, was analyzed. In addition, two unpublished datasets (Schedin-Weiss *et al*. in preparation) from our research group were included in this study, as they met with the above-mentioned inclusion criteria (for pre-processed datasets, see^20^). Lastly, studies using pooled biological samples or performing targeted MS approach were excluded from the meta-analysis.

Neuropathological evaluation of neurofibrillary tangles (Braak staging)^54^ and neuritic plaques (CERAD criteria)^55^ was done in all postmortem human brain tissue included in the above-mentioned studies. While AD cases had often higher Braak stages (IV-VI), control subjects presented little or no pathological alterations beyond normal age-appropriate changes (0-III). Cases with non-AD pathological changes were reported to be excluded in these studies.

### Data pre-processing

The protein identifiers were manually set to UniProtKB accession numbers in each dataset. Each dataset was standardized in the same manner by allowing 20% of missing values per group, converting protein intensities to log2 scale, applying median-centered normalization and scaling (median 0 and standard deviation 1) so that they would be comparable for further statistical analyses. Subsequently, sample distributions were visually inspected by PCA for each dataset as well as for the concatenated data, consisting of 533 proteins quantified in all 857 samples and all 18 datasets. All analyses were performed in R (version 4.0.1).

### Meta-analysis by random-effects-model

The meta-analysis was performed using the meta package (version 4.13) and the metacont function^56^. A random-effects-model, using the DerSimonian and Laird method^57^, was selected, due to methodological differences noted between the selected studies, such as clinical characteristics of postmortem human samples, brain region, sample preparation and LC-MS/MS experiments (Online-only Tables 1 and 2). For each protein, the effect size (i.e., mean difference between AD and control) was computed by taking into account the following parameters: mean of protein intensities per protein per group, standard deviation of protein intensities per protein per group and sample size per group. Particularly, this allowed accounting for group differences even when proteins showed opposite directional changes between datasets or had not been quantified in all datasets. The p-values were corrected for multiple hypothesis testing using Benjamini-Hochberg method and considered significant at ≤10% FDR.

### Gene set analysis

To gain better insights into the biological processes underlying AD pathogenesis, GSA was performed using the piano R package^21^. Most of the selected proteomic studies were protein-centric, thereby containing the isoform-specific information. Prior to GSA, proteins from the meta-analyses were converted to genes, giving rise to multiple values for the same gene. If isoforms showed consistent mean difference in AD, those with the least significant changes were then excluded from GSA. On the other hand, all isoforms that did not follow the same direction of change were excluded from GSA. Thus, a reduced number of genes were subjected to GSA, compared to the protein list obtained from the meta-analysis^20^. Subsequently, the UniProtKB accession numbers were converted to gene symbol using the UniProt Retrieve/ID mapping tool. All genes together with mean difference and p-value, which were computed by the random-effects-model, were subjected to GSA, thus allowing for a comparison between significant alterations and all identified genes (background) from our meta-analysis data. Two databases, KEGG 2019 and GO biological process, were used as retrieved from Enrichr^58,59^. Minimum number of genes associated with a given geneset was set to five. To get a better understanding of how pathways could be dysregulated in AD, distinct upregulation and distinct downregulation categories were selected. Pathways with FDR < 5% were considered as statistically significant.

## Data Availability

The raw data of the following datasets can be found at the ProteomeXchange Consortium via the PRIDE partner repository or the Synapse Web Portal: Dataset 1 (PXD014557)^14,60^, Dataset 4 (PXD006122)^8,61^, Datasets 5–8 (PXD010138)^19,62^, Dataset 9 (PXD007160)^12,63^, Dataset 10 (syn16816734)^10,64^, Dataset 11 (syn21441771)^6,65^, Dataset 12 (syn21441782)^6,66^, Dataset 13 (syn21444768)^6,67^, Datasets 14–15 (PXD007985)^9,68^, Dataset 17 (PXD008739)^11,69^, and Dataset 18 (PXD008806)^11,70^. In addition, Dataset 16 was retrieved from the supplementary data provided in the original publication^7^. Lastly, pre-processed data from Datasets 2 and 3 are available at the figshare database^20^. To support data sharing and reusability, all fully processed individual datasets are available in github, which can be accessed by https://github.com/hazhay/Meta-analysis_AD. In addition, input datasets used for meta-analyses and for GSAs as well as their findings are available at the figshare database associated with this article^20^.

## Online-only Tables

**Online-only Table 1:** Studies selected for meta-analysis.

Dataset	Reference	Brain region	MS approach	Lysis buffer	Sample size	Age (mean)	PMI (h) (mean)	Gender (Female/Male)	APOE4 status	Braak stage	Number of proteins^*^
1	*Haytural et al. Mol Cell Proteomics, 2020* ^14^	Outer 2/3 of the molecular layer (Dentate gyrus)^#^	TMT	SDS-based	5 AD	91 ± 4.3	5.17 ± 1.3	4 F / 1 M	2/5	IV	7458
					5 C	81.8 ± 9.2	6.8 ± 1	4 F / 1 M	1/5	0-II	
2	*Schedin-Weiss et al. (manuscript in preparation)*	Frontal cortex^‡^	^18^O	Urea-based	5 AD	81 ± 5	6.3 ± 1.1	3 F / 2 M	n/a	III – VI	2298
					5 C	79 ± 6	6.8 ± 2	3 F / 2 M		0-II	
3		Hippocampus^#^			5 AD	81 ± 5	6.3 ± 1.1	3 F / 2 M	n/a	III – VI	2251
					5 C	79 ± 6	6.8 ± 2	3 F / 2 M		0-II	
4	*Bereczki et al. Brain, 2018* ^8^	Dorsolateral prefrontal cortex (BA9)^‡^	TMT	SDS-based	9 AD	85.6 ± 6.5	23.7 ± 10	6 F / 3 M	n/a	-	7097
					8 C	83 ± 3.8	35.2 ± 18.4	4 F / 4 M			
5	*Mendonça et al. Neurobiol Dis, 2019* ^19^	Entorhinal cortex^#^	Label-free	Urea-based	6 AD	82.8 ± 5.19	4.58 ± 1.5	4 F / 2 M	n/a	IV – VI	2014
					7 C	65 ± 10.2	4.36 ± 2.43	1 F / 6 M		n/a	
6		Frontal cortex^‡^			10 AD	82.5 ± 6.06	5.25 ± 0.89	8 F / 2 M	n/a	IV – VI	2230
					7 C	58.3 ± 12.7	5.29 ± 3.4	2 F / 5 M		n/a	
7		Parahippocampal cortex^#^			5 AD	80.2 ± 9.42	4.9 ± 0.74	5 F	n/a	V – IV	2204
					11 C	64.1 ± 14.1	4.77 ± 2.79	4 F / 7 M		n/a	
8		Temporal cortex^#^			11 AD	81.2 ± 8.05	5 ± 1.28	9 F / 2 M	n/a	IV – VI	2101
					7 C	58.3 ± 12.7	5.29 ± 3.4	2 F / 5 M		n/a	
9	*Ping et al. Sci Data, 2018* ^12^	BA9^‡^	TMT	Urea-based	10 AD	65.1 ± 7.43	5.4 ± 1.5	5 F / 5 M	7/10	V – VI	7129
					10 C	63.7 ± 10.2	9.3 ± 7.09	5 F / 5 M	2/10	0 - II	
10	*Johnson et al. Mol Neurodegener, 2018* ^10^	BA9^‡^	TMT	Urea-based	20 AD	86.3 ± 8.7	14.2 ± 4.9	10 F / 10 M	7/20	IV – VI	5888
					13 C	81.3 ± 10.9	17.4 ± 5.8	3 F / 10 M	1/13	I-III	
11	*Johnson et al. Nat Med, 2020* ^6^	BA9^‡^	Label-free	Urea-based	252 AD	-	5.8 ± 4.6	141 F / 111 M	n/a	III – VI	2793
					94 C	-	8.2 ± 6.6	44 F / 50 M		0 - III	
12		Temporal cortex^#^			83 AD	82.3 ± 7.7	7.6 ± 4.8	47 F / 36 M	43/83	IV – VI	3286
					28 C	89 ± 1.4	11.3 ± 8.7	17 F / 11 M	3/28	0 - III	
13		BA9^‡^			49 AD	78.1 ± 10.7	12.7 ± 5.4	27 F / 22 M	n/a	?	2249
					46 C	65.2 ± 8.9	14.7 ± 4. 9	19 F / 27 M		?	
14	*Bai et al. Neuron, 2020* ^9^	Frontal gyrus^‡^	TMT	Urea-based	15 AD	78 ± 8.5	3.1 ± 0.92	9 F / 6 M	n/a	V – VI	12521
					18 C	84.9 ± 5.9	2.6 ± 0.7	8 F / 10 M		I - IV	
15		Frontal gyrus^‡^			39 AD	84 ± 10.1	6.4 ± 4.4	27 F / 12 M	22/39	II – VI	12147
					23 C	80.8 ± 10.4	9.5 ± 6.3	11 F / 12 M	2/23	0 - III	
16	*Zhang et al. Acta Neuropathol Commun., 2018* ^7^	Frontal cortex^‡^	Label-free	SDS-based	8 AD	70.8 ± 5.84	7.2 ± 4.06	2 F / 6 M	4/8	V – VI	1968
					8 C	70.8 ± 11.8	7.5 ± 2.6	4 F / 4 M	1/8	0 - II	
17	*Xu et al. Commun Biol., 2019* ^11^	Hippocampus^#^	iTRAQ	SDS-based	6 AD	70.6 ± 7.8	6.5 ± 1.3	3 F / 3 M	n/a	V – VI	2910
					6 C	69.1 ± 7.3	10.2 ± 3.1	3 F / 3 M		n/a	
18		Entorhinal cortex^#^			9 AD	70.3 ± 7	6.8 ± 2.3	4 F / 5 M	n/a	IV – VI	2585
					9 C	70.1 ± 6.6	9.6 ± 2.6	4 F / 5 M		n/a	
**Concatenated data (input for meta-analysis)**					**Sample size**						**Number of proteins**
Labeled data					123 AD						17296
					102 C						
Label-free data					424 AD						4292
					208 C						

^*^Number of proteins, after allowing 20% of missing values, were reported for each study.

^#^Studies comprising temporal lobe.

^‡^Studies comprising frontal lobe.

**Online-only Table 2:** Methodological differences between the studies.

Reference	Brain bank	Protein digestion	Sample clean-up	Mass spectrometer	Pre-fraction technique	Database search engine	Protein database
*Haytural et al. Mol Cell Proteomics, 2020* ^14^	Netherlands Brain Bank	Lys-C and Trypsin	Sera Mag SP3	Q Exactive mass spectrometer (Thermo Scientific)	High resolution iso-electric focusing (72 fractions)	MSGF+	Ensembl 75
*Schedin-Weiss et al. (manuscript in preparation)*	Netherlands Brain Bank	Trypsin	ZipTip C_18_	Q Exactive mass spectrometer (Thermo Scientific)	SCX ion exchange (4 fractions)	Proteome Discoverer	Swiss-Prot
*Bereczki et al. Brain, 2018* ^8^	The Brains for Dementia Research	Lys-C and Trypsin	FASP	Q Exactive mass spectrometer (Thermo Scientific)	High resolution iso-electric focusing (72 fractions)	MSGF+	Swiss-Prot
*Mendonça et al. Neurobiol Dis, 2019* ^19^	Human Brain Tissue Bank from Semmelweis University, Budapest	Trypsin	FASP	Q Exactive mass spectrometer (Thermo Scientific)	No	Proteome Discoverer	Swiss-Prot
*Ping et al. Sci Data, 2018* ^12^	Emory Alzheimer’s Disease Research Center Brain Bank	Lys-C and Trypsin	Sep-Pak C18 cartridge	Orbitrap Fusion mass spectrometer (Thermo Scientific)	Electrostatic repulsion-hydrophilic interaction chromatography (21 fractions)	Proteome Discoverer	Swiss-Prot TrEMBL
*Johnson et al. Mol Neurodegener, 2018* ^10^	Baltimore Longitudinal Study of Aging (BLSA)	Lys-C	Sep-Pak C18 cartridge	Orbitrap Fusion mass spectrometer (Thermo Scientific)	Electrostatic repulsion-hydrophilic interaction chromatography (20 fractions)	Proteome Discoverer	Swiss-Prot TrEMBL
*Johnson et al. Nat Med, 2020* ^6^	BLSA, Mount Sinai School of Medicine Brain Bank, Banner Sun Health Research Institute, Adult Changes in Thought Study	Lys-C and Trypsin	Sep-Pak C18 cartridge	Q Exactive mass spectrometer (Thermo Scientific)	No	MaxQuant	Swiss-Prot TrEMBL
	Mayo Clinic Brain Bank						
	University of Pennsylvania School of Medicine Brain Bank						
*Bai et al. Neuron, 2020* ^9^	Brain and Body Donation Program at Banner Sun Health Research Institute	Lys-C and Trypsin	Sep-Pak C18 cartridge	Q Exactive mass spectrometer (Thermo Scientific)	Basic pH HPLC (40–120 fractions)	Jump Suite including SEQUEST and MASCOT	Swiss-Prot TrEMBL UCSC
	Alzheimer’s Disease Research Center at Icahn School of Medicine at Mount Sinai						
*Zhang et al. Acta Neuropathol Commun., 2018* ^7^	Emory Center for Neurodegenerative Disease Brain Bank	Trypsin	FASP	LTQ Orbitrap Elite (Thermo Scientific)	No	Proteome Discoverer	TrEMBL (reviewed entries)
*Xu et al. Commun Biol., 2019* ^11^	New Zealand Neurological Foundation Human Brain Bank	Trypsin	—	QSTAR Elite Q-TOF spectrometer (AB SCIEX)	Basic pH HPLC (86 fractions)	Protein Pilot	Swiss-Prot

## Code availability

The R script used in this research is publicly available and can be found in github (https://github.com/hazhay/Meta-analysis_AD).
